# Relation between high levels of myeloperoxidase in the culprit artery and microvascular obstruction, infarct size and reverse remodeling in ST-elevation myocardial infarction

**DOI:** 10.1371/journal.pone.0179929

**Published:** 2017-07-13

**Authors:** Karim Stamboul, Marianne Zeller, Luc Rochette, Yves Cottin, Alexandre Cochet, Thibault Leclercq, Guillaume Porot, Charles Guenancia, Marie Fichot, Nicolas Maillot, Catherine Vergely, Luc Lorgis

**Affiliations:** 1 Department of Cardiology, University Hospital, Bd de Lattre de Tassigny, Dijon Cedex, France; 2 MRI Unit and LE2I UMR CNRS 6306, University Hospital, Dijon, France; 3 Laboratory of Cerebro-Vascular Pathophysiology and epidemiology (PEC2), University of Burgundy, Dijon, France.University of Burgundy, Dijon, France; University of Bologna, ITALY

## Abstract

**Main objective:**

To better understand the role of myeloperoxidases (MPO) in microvascular obstruction (MO) phenomenon and infarct size (IS) using cardiac magnetic resonance (CMR) data in patients with acute myocardial infarction (AMI).

**Method:**

40 consecutive patients classified according to the median level of MPO in the culprit artery. A CMR study was performed during the week following AMI and at 6 months, with late gadolinium enhancement sequences.

**Results:**

Persistent MO was observed in the same proportion (50 vs. 65%, p = 0.728) between the low vs. high MPO group levels. However, the extent of the microvascular obstruction was significantly greater in the high-MPO group (6 (0–9) vs.16.5 (0–31), p = 0.027), together with a greater infarct size, and a trend towards a lower left ventricular ejection fraction (LVEF) (p = 0.054) at one week. CMR data at 6 months showed that reverse systolic remodeling was two fold more present in the low-MPO group (p = 0.058). Interestingly, the extent of MO (8.5 (6.5–31) vs. 4.1 (3–11.55), p = 0.042) and IS remained significantly greater (24.5 (9.75–35) vs. 7.5 (2.5–18.75), p = 0.022) in the high-MPO group. Moreover, MPO in the culprit artery appeared to correlate positively with MPO in non-culprit arteries and serum, and with troponin levels and peak CK.

**Conclusion:**

This patient-based study revealed in patients after AMI that high MPO levels in the culprit artery were associated with more severe microvascular obstruction and greater IS. These findings may provide new insights pathophysiology explanation for the adverse prognostic impact of MO.

## Introduction

Myeloperoxidase (MPO) is a member of the heme peroxidase superfamily. It is secreted from activated neutrophils, monocytes and certain tissue macrophages, and generates reactive oxygen species and diffusible radical species [1]. Studies have shown that MPO levels are a predictor of death after chest pain [2] and acute myocardial infarction [3]. However, the mechanisms by which MPO adversely affects the prognosis are only partly understood. In addition to its pathophysiological role in atherogenesis [4], MPO has recently been shown to contribute to microvascular obstruction in AMI patients [5]. During myocardial ischemia-reperfusion sequences, microvascular function is associated with recruitment of polymorphonuclear neutrophils (PMN) and has been attributed to decreased bioavailability of nitric oxide (NO). Endothelium-dependent microvascular function is a multifactorial process involving endothelial injury or dysfunction, neutrophil accumulation, the over-production of reactive oxygen species, thrombus embolization and activation of the coagulation cascade [6]. Among different tools used to assess the microvascular obstruction, cardiac magnetic resonance imaging (CMR) with a gadolinium-based contrast agent shows the extent of myocardial damage following infarction, areas of hyperenhancement on late gadolinium-enhanced (LGE) images (= infarct size) and left ventricular remodeling indexes [7]. Moreover, regions of persistent hypoenhancement in the core of the infarcted myocardium have been shown to reflect no reflow [8]. One of the most important pathogenic pathways involving MPO might be endothelial dysfunction. Considering the strong relation between MPO and systemic endothelial function, it might also be speculated that systemic activation of leukocytes, particularly via secretion of MPO, is one mechanism by which AMI leads to systemic endothelial dysfunction [9]. It has been suggested that both endothelial function and LDL/HDL oxidation due to MPO release are involved in the occurrence of NR and its consequences such as infarct size (IS) enlargement and left ventricular (LV) remodeling. The objectives of our study were to assess plasma MPO levels, and endothelial dysfunction witnessed by plasma levels of L-arginine and its methylated derivatives, asymmetric and symmetric dimethylarginine (ADMA and SDMA) at admission in first ST elevation myocardial infarction (STEMI) patients, and to explore the relationship between such biomarkers with microvascular obstruction, IS and LV remodeling by cardiac magnetic resonance (CMR) at 3 days and 6 months.

## Patients and methods

The participants in this study were recruited from the observatoiRe des Infarctus de Côte d’Or (RICO) survey, a French regional survey for AMI, the details of which have been previously published [10]. In the present study, we design a patient-based study including male patients admitted with a first acute ST-Elevation MI within 12 hours after symptom onset were included. MI was diagnosed according to European Society of Cardiology and American College of Cardiology criteria [11]. All patients were treated by a primary or rescue PCI procedure with thrombectomy. Patients under 18 years of age, patients with cardiogenic shock, acute infection, acute inflammation, severe renal impairment, or with a prior chronic treatment (or for at least 7 days) with vitamin C, steroids or non-steroid anti-inflammatory drugs, and those with a CRP> 30 mg/l were excluded from the study. Forty-nine patients with AMI were recruited during the study period. Five were excluded because of a contra-indication to CMR (mainly claustrophobia), and 4 because of incomplete biological data. This study complied with the Declaration of Helsinki, was approved by the ethics committee of the University Hospital of Dijon, and each patient gave written consent before participation.

### Data collection

The data included demographics, cardiovascular risk factors (history of hypertension or treated hypertension, diabetes, history of hypercholesterolemia or treated hypercholesterolemia, current smoking), admission characteristics and hemodynamic parameters. Patients’ BMI (weight (kg)/height (m^2^)) was measured within 48 hours of admission. Ischemic time was the time between symptom onset and crossing the culprit lesion with the floppy guide wire. We recorded anterior wall infarction, and coronary angiograms before intervention, after aspiration thrombectomy, and at the end of the procedure were analyzed. We evaluated baseline, post-thrombectomy, and final antegrade coronary flow according to the TIMI (Thrombolysis In Myocardial Infarction) criteria, together with the final myocardial blush grades. No reflow was defined as TIMI flow grade ≤2 or TIMI flow grade 3 with myocardial blush grade ≤1 at the final angiogram [12]. Blood samples were drawn at admission. We assessed peak plasma CK by sampling every eight hours during the first two days after admission. Plasma creatinine levels were measured on a Vitros 950 analyzer (Ortho Clinical Diagnostics, Rochester, NY). C-reactive protein (CRP) was measured on Dimension Xpand (Dade Behring, Newark, Neb) with an immunonephelometry assay. Plasma N-terminal pro B-type natriuretic peptide (NT-proBNP) was determined by ELISA with an Elecsys NT-proBNP sandwich immunoassay on Elecsys 2010 (Roche Diagnostics, Basel, Switzerland). Total cholesterol (TC), high-density lipoprotein cholesterol (HDL-C) and triglyceride (TG) concentrations were measured on a Dimension analyzer (Dade Behring, Newark, NE). The level of low-density lipoprotein cholesterol (LDL-C) was calculated using the Friedewald formula. Plasma glucose concentrations (enzymatic method (glucose oxidase)) and creatinine levels were measured on a Vitros 950 analyzer (Ortho Clinical Diagnostics, Rochester, NY) and creatinine clearance was calculated with the Cockcroft formula. Glycated hemoglobin A1c (HbA1c) was measured with ion exchange HPLC (Bio-Rad Laboratories, Richmond, CA).

### Oxidative stress, endothelial function and inflammation markers

Plasma myeloperoxidase levels, reactive oxygen species (ROS) assay, plasma antioxidant activity, L-arginine, ADMA and SDMA were determined at 3 sites: 1) **venous blood samples** and other blood samples taken before angioplasty and stenting from the 2) **coronary ostium of a non-culprit artery** through the guiding catheter and from 3) **the culprit artery** at the culprit lesion site by the Export^®^ thrombectomy catheter. It consisted of a 6-Fr rapid exchange catheter (1.37 mm internal lumen) and vacuum syringe connected to its proximal end. The distal tip was advanced along the guide wire up to the thrombus and then the vacuum syringe was opened to aspirate the thrombus from the coronary artery. Samples were centrifuged at 2000 x g for 10 minutes at 4°C. The supernatants were decanted and frozen at -80°C until analysis.

Plasma MPO concentrations were measured by quantitative sandwich enzyme immunoassay (Human MPO, Quantikine^®^ ELISA, R&D Systems, Abingdon, UK). ROS were determined using the free oxygen radical monitor (FORM, FORM-PLUS-3000, Optimabio, France) and kit. The FORM indirectly measures the concentration of hydroperoxides by a colorimetric assay using Free Oxygen Radical Testing (FORT) as previously described [13]. The ROS concentration is reported in FORT units, where 1 FORT unit corresponds to 7.6μmol/L (0.26 mg/L of H_2_O_2_). Total plasma Oxygen Radical Antioxidant Capacity (ORAC) was determined by the method first described by Cao et al. as detailed [14]. The method is based on the ability of plasma components to protect an indicator protein, allophycocyanin (APC), whose fluorescence is altered when it is oxidized. The results are expressed as ORAC units, where 1 ORAC unit equals the net protection provided by 1 μmol/L Trolox.

L-arginine, ADMA, and SDMA were measured by high performance liquid chromatography (HPLC) as previously described [15]. The detection limits were 0.05 and 1.19 μmol/L and inter-day variabilities were 5.7 and 4.6% for ADMA and L-arginine, respectively.

### CMR protocol

Patients underwent CMR a mean of 3 days (3±2 days) after admission and 6 months after AMI. CMR was performed on a 3.0-T Magnet (Trio TIM; Siemens Medical Solutions, Germany), using a phased-array thoracic coil. Patients who did not benefited from two CMRI examinations were excluded from the study. In all sequences, the signal intensity correction algorithm provided by the manufacturer was applied. The patient was placed in the supine position and images were acquired under cardiac gating. To evaluate left ventricular function, cine-CMR was performed using a breath-hold, ECG-gated trueFISP sequence, (TR/TE 3.4/1.7 ms, FA 57°, FOV 350x420 mm^2^, acquisition matrix 150x192). A series of short-axis slices (thickness 5 mm, interslice gap 5 mm) was defined from the base of the heart to the apex. To evaluate microvascular obstruction and infarct size, Late Gadolinium-Enhanced (LGE) images were acquired 10 minutes after a bolus injection of 0.1 mmol/Kg Gd-DOTA into a brachial vein. A segmented T1-weighted Phase-Sensitive Inversion Recovery (PSIR) sequence was used (TR/TE/TI: 3.5/1.42/400 ms, FA 20°, matrix 175x256, FOV 240x350 mm^2^, pixel size 1.37x1.37 mm^2^; acquisition time of 10 to 15 seconds according to the RR; cardiac gating every second cycle). The image planes were the same as for the functional and perfusion studies (thickness of 8 mm with an interslice gap of 2 mm). Horizontal and vertical long-axis slices were also obtained. The sequence parameters were: repetition time/echo time = 780/1.56 ms, flip angle = 10, field of view = 350x400 cm, and matrix = 128x256, in plane resolution = 2.3x1.2. The inversion time was 270–325 ms (set to null normal myocardium). The end-diastolic phase was chosen to set the acquisition window. The images at the end-systole and end-diastole were taken into account.

### CMR data analysis

Prospective image analyses were performed using QIR^®^ software (Université de Bourgogne, Dijon, France). Left ventricular End-Diastolic Volume (EDV) and End-Systolic Volume (ESV) were calculated from short-axis views. The Left Ventricular Ejection Fraction (LVEF) was calculated with the following formula: LVEF = (EDV-ESV)/EDV. Reverse remodeling was defined as a decrease in LVESV >10% on the cardiac MR images obtained one week and 6 months after STEMI [16]. For the detection and quantification of the infarct zone and microvascular obstruction, an automatic method was used. First, the endocardial and epicardial contours were manually traced on each short-axis LGE image. The myocardium area was segmented as normal and pathological areas using a Gaussian mixture model (GMM).

Microvascular obstruction was defined as a dark area surrounded by the MI area. Infarct size (IS) was determined for each patient by summing the results for each slice multiplied by the gap between slices, and was expressed as a percentage of total myocardial volume. In addition, delayed enhancement images were visually analyzed according to a 17-segment model. Images were analyzed by the consensus of 2 expert observers, blinded to patient data and the results of other examinations. The inter-observer variability was less than 5%. In cases of disagreement, the final decision was taken by consensus.

## Statistical analysis

Patients were dichotomized into two groups according the median MPO levels in the culprit artery (median value = 640 ng/mL.). The results are expressed as median values (25th-75th percentile) for continuous variables or as percentages for qualitative variables. The normality of distribution for each variable was analyzed by the Kolmogorov-Smirnov test. The Spearman or Pearson test was used to determine correlations between continuous variables and MPO values. For binary variables, the median MPO levels were compared with a Mann-Whitney test. A multiple linear regression analysis was performed to determine the independent factors associated with MPO levels. All variables listed in Table 1 were tested in univariate analysis and were introduced into the multivariate model if the p value<0.20. All analyses were performed using Sigmastat Software (Jandel Inc.).

**Table 1:** Patients’ characteristics. Data are presented as n (%) or median (25th-75th).

	Low MPO N = 20	High MPO N = 20	p
Age, *years*	59(48–66)	54(48–66)	0.766
BMI, *kg/m*^*2*^	25.50(23.25–28.00)	26(24.25–28.00)	0.599
Waist circumference, *cm*	96(86.25–103)	96.50(90.00–103.25)	0.841
Hypertension,	5(25)	4(20)	0.705
Hypercholesterolemia	8(40)	4(20)	0.168
Family history of CAD	1(5)	0	0.311
Diabetes	0	2(10)	0.147
Smoking	13(65)	9(45)	0.204
**Clinical data**			
SBP, *mmHg*	132.5(118.0–147.5)	128.0(116.5–148.0)	0.903
LVEF, *%*	55(46–60)	53(36–60)	0.178
LVEF>40%	18(56)	14(43)	0.114
GRACE risk score	82(60–104)	74(65–106)	0.924
Delay>120 min	5(45.5)	6(54.5)	0.383
Previous angina	13(46)	15(54)	0.490
KILLIP adm>1	1(25)	3(75)	0.292
Anterior wall location	8(40)	13(65)	0.113
Thrombolysis	7(34)	3(16)	0.200
PPCI	12(60)	14(78)	0.239
Delay symptoms-reperfusion, *min*	190(112.5–360)	180(115–355)	0.957
STR>50%	16(94)	15(83)	0.316
Post-PCI No-reflow in angio TIMI	1(5)	3(16)	0.267
**Biological data**			
HDL, *mmol/L*	0.38(0.35–0.41)	0.47(0.40–0.56)	0.017
LDL, *mmol/L*	1.44(1.15–1.60)	1.58(1.19–1.77)	0.267
TC, *mmol/L*	2.23(1.83–2.35)	2.24(1.94–2.47)	0.423
Log CK	3.10(2.73–3.42)	3.60(3.36–3.74)	0.003
Log BNP	1.85(1.67–2.47)	2.05(1.73–2.83)	0.308
Glycemia, *mmol/L*	6.85(6.31–8.17)	7.13(6.14–8.44)	0.803
HbA1C, *%*	5.70(5.50–6.00)	5.95(5.53–6.23)	0.181
CRP, *mg/L*	3.95(2.99–11.25 )	3.00(2.92–5.75)	0.545
Creat cl, *mL/min*	104(78–122)	98(77–120)	0.963
MPO nc, ng/ml	494.23(380.76–658.59)	810.25(672.54–997.59)	0.001
MPO vn, ng/ml	418.02(302.50–546.54)	769.82(425.74–1075.22)	0.003
ADMA ca, *μmol/L*	0.31(0.26–0.38)	0.34(0.28–0.41)	0.552
ADMA nc, *μmol/L*	0.35(0.30–0.43)	0.37(0.30–0.45)	0.818
ADMA vn, *μmol/L*	0.42(0.36–0.52)	0.42(0.35–0.49)	0.818
L-arg ca, *μmol/L*	76.4(60.8–84.7)	57.4(46.7–74.6)	0.023
L-arg nc, *μmol/L*	83.1(69.3–98.2)	57.96(51.1–73.6)	0.001
L-arg vn, ***μ****mol/L* SDA	65.7(56.0–105.4)	70.9(61.2–89.1)	0.808
SDMA ca, *μmol/L*	0.26(0.24–0.37)	0.34(0.28–0.38)	0.062
SDMA nc, *μmol/L*	0.31(0.26–0.41)	0.35(0.31–0.44)	0.144
SDMA vn, *μmol/L*	0.44(0.36–0.52)	0.42(0.39–0.52)	0.903
L-arg/ADMA ca	209.07(178.01–256.74)	174.71(144.57–233.81)	0.746
L-arg/ADMA nc	215.14(182.15–276.11)	169.10(130.98–206.80)	0.275
L-arg/ADMA vn	180.82(133.93–240.17)	182.20(133.85–206.01)	0.387
FORT ca, *μmol/L*	491.69(451.81–562.08)	447.23(358.00–684.00)	0.112
FORT nc, *μmol/L*	496.92(419.77–574.08)	486.00(329.54–580.00)	0.695
FORT vn, *μmol/L*	491.85(383.31–558.73)	450.00(358.00–684.00)	0.918
ORAC ca, *μmol/L*	1.92(1.73–2.31)	1.79(1.34–2.88)	0.689
ORAC nc, *μmol/L*	2.44(1.91–2.87))	1.99(1.61–4.32)	0.739
ORAC vn, *μmol/L*	1.91(1.59–2.24)	2.02(1.50–3.58)	0.641

ca, culprit artery; nc, non-culprit artery; vn, venous blood

## Results

Our study included forty patients who were divided into 2 groups according to the median MPO level, which was 639.63ng/mL. Baseline demographic and clinical characteristics in the low and high MPO level groups are shown in Table 1. There were no statistical differences between the groups for age, baseline demographics, cardiovascular risk factors and clinical characteristics. Unrecognised non-Q-wave MI was present in the same proportion in both groups (p = 0.205). Also, there were no significant differences between the 2 groups for the hemodynamic parameters on admission. Chronic medications prior to the MI (aspirin, B-blockers) and acute pharmacological management (unfractionated heparin, low molecular weight heparin, acetylsalicylic acid, clopidogrel loading dose, abciximab infusion) were strictly identical in both groups. Furthermore, anterior wall location, median time to reperfusion, the number of diseased vessels and baseline TIMI flow in the culprit artery were similar in both groups.

### MPO, MO and infarct size

**Fig 1:**
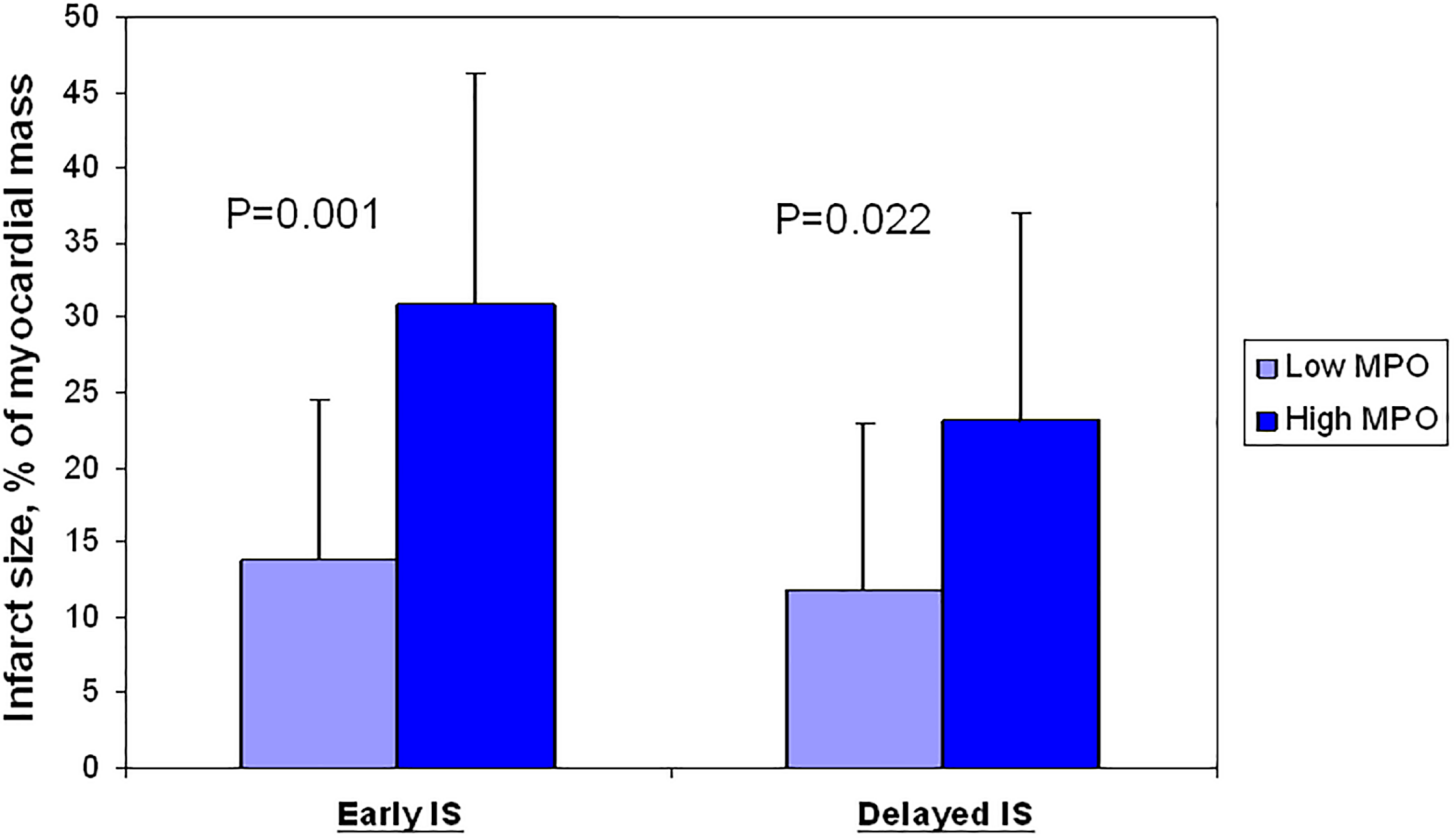
Infarct size regarding MPO plasma levels in the culprit artery assessed by CMR at day 3 and 6 months.

Microvascular obstruction as assessed by CMR at day 3 was observed in the same proportion (50 vs. 65%, p = 0.728) between the low vs. high MPO group levels. However, the extent of the microvascular obstruction was significantly greater in the high-MPO group (6 (0–9) vs.16.5 (0–31), p = 0.027) (Table 2), together with a greater infarct size, and a trend towards a lower left ventricular ejection fraction (LVEF) (p = 0.054) at one week. CMR data at 6 months showed that reverse systolic remodeling was two fold more present in the low-MPO group (p = 0.058). Interestingly, the extent of MO (8.5 (6.5–31) vs. 4.1 (3–11.55), p = 0.042) and IS remained significantly greater (24.5 (9.75–35) vs. 7.5 (2.5–18.75), p = 0.022) (Fig 1) in the high-MPO group. Plasma MPO levels at the culprit artery positively correlated with the IS at the acute phase but also at 6-months (respectively r = 0.497, p = 0.003 and r = 0.502, p = 0.006). Despite similar results were obtained with MPO at the ostium of the non-culprit coronary artery (r = 0.358, p = 0.038 and r = 0.454, p = 0.015), the coefficient of correlation were less good, and comparison of IS on CMR after dichotomization to the median of MPO didn’t reach the significance. Finally, MPO levels in the culprit artery appeared to correlate positively with MPO levels in both non-culprit arteries and serum, and with troponin levels and peak CK (Table 3). Multiple linear regression analysis showed that Peak CK (p = 0.012), MPO in non culprit artery (p<0.001) and the neutrophil count (p = 0.039) were independent predictors of MPO values. This statistical model can explain 44% of the variance in MPO values (R^2^ = 0.44) (Table 4).

**Table 2:** CMR data at acute phase and at 6 months.

Acute phase	Low MPO Median <640 ng/mL N = 20	High MPO Median >640 ng/mL N = 20	p
LEVF, %	56(48–60)	49(39–54)	0.054
EDV index, mL	88(72.5–122.5)	95(92.5–102)	0.294
ESV index, mL	43(27.5–59)	52(42.5–58.5)	0.151
Presence of MO	10(50)	13(65)	0.728
Extent of MO, %	6(0–9)	16.5(0–30)	0.027
Infarct Size, %	12(6–22)	31(16–44)	0.001
**6-month CMR**			
LEVF, %	63(55–69)	52(44–66)	0.064
EDV index, mL	87(76–116)	97(85–114)	0.315
ESV index, mL	29(25–52)	47(28–67)	0.109
Presence of MO	2(10)	3(15)	1.000
Extent of MO, %	4.1 (3–11.55)	8.5 (6.5–31)	0.042
Infarct Size, %	7.5(2.5–18.75)	24.50(9.75–35)	0.022
Systolic remodeling	6(30)	13(65)	0.066
Reverse remodeling	12(60)	6(30)	0.058

EDV, end-diastolic volume; ESV, end-systolic volume; IS, infarct size; LVEF, left ventricular ejection fraction; MO, microvascular obstruction.

**Table 3:** Determinants of the MPO value in univariate regression (n = 40).

	MPO culprit artery		MPO non-culprit artery		MPO serum	
	r	p	r	p	r	p
MPO culprit artery	***	***	0.707	<0.001	0.592	<0.001
MPO non-culprit artery	0.707	**<0.001**	***	***	0.633	<0.001
MPO serum	0.592	**<0.001**	0.633	<0.001	***	***
ADMA culprit artery	0.083	0.639	0.016	0.929	-0.251	0.159
ADMA non-culprit artery	0.054	0.761	0.170	0.336	-0.225	0.208
ADMA serum	-0.027	0.879	0.152	0.392	-0.050	0.782
SDMA culprit artery	0.073	0.682	0.204	0.246	-0.106	0.558
SDMA non-culprit artery	0.013	0.942	0.301	0.084	-0.139	0.441
SDMA serum	-0.190	0.282	0.023	0.896	-0.224	0.210
L-arg culprit artery	-0.181	0.305	-0.457	**0.007**	-0.611	**<0.001**
L-arg non-culprit artery	-0.422	**0.013**	-0.450	**0.008**	-0.478	**0.005**
L-arg serum	-0.013	0.941	-0.013	0.943	-0.098	0.589
CRP	-0.063	0.725	0.040	0.823	-0.117	0.516
Nt-proBNP	0.172	0.340	0.220	0.220	-0.019	0.918
Creatinine clearance	0.183	0.301	0.002	0.992	0.267	0.133
Troponin	0.450	**0.008**	0.287	0.107	0.177	0.325
Neutrophil Count	0.188	**0.009**	0.200	**0.023**	0.258	**0.001**
Peak CK	0.437	**0.010**	0.490	0.003	0.252	0.157

***NA

**Table 4:** Determinants of the MPO value (culprit artery) by multiple linear regression (n = 40).

	Total Population	
	Coefficient β	p
Peak Creatinine kinase	+0.198	0.012
MPO non culprit	+0.172	<0.001
Age	+0.133	0.101
Neutrophil Count	+0.074	0.039
LVEF > 40%	-0.134	0.265
R^2^	0.44	

LVEF: Left Ventricular Ejection Fraction.

### MPO, L-arginine and derivatives, plasma antioxidant activity assessment

Comparing the two groups, we found no significant difference for the FORT and ORAC evaluations (Table 1). Conversely, intra-coronary plasma L-arginine levels (in culprit and non-culprit arteries) were significantly lower in the high-MPO group (respectively 57.4 vs. 76.4 *μ*mol/L; p = 0.023 and 58.0 vs. 83.1 *μ*mol/L; p = 0.001). Interestingly, there was also a trend towards a higher plasma level of SDMA in the culprit artery in the high-MPO group (0.34 vs. 0.26 *μ*mol/L; p = 0.062). There was no difference between these two groups regarding ADMA levels (Table 1). Nitric oxide bio-availability assessed by the L-arg/ADMA ratio at the culprit lesion site, showed that there was no difference for this parameter between the low and high-MPO group. However, patients with high MPO levels had a higher plasma HDL level (0.47 mmol/L vs. 0.38 mmol/L).

## Discussion

In this study, we demonstrated an interaction between MPO levels on admission and extent of microvascular obstruction, IS and systolic LV remodeling as assessed by the gold-standard technique during acute phase, but also in the middle term follow-up. Our data highlighted the pathophysiological role of neutrophils during myocardial injury and the potential involvement of MPO in the pathophysiology of this disease, together with his consequences on the ischemic myocardium. Myeloperoxidase is a heme enzyme abundantly expressed in PMN and accounts for up to 5% of the dry weight of these cells [17]. Although MPO is also expressed in monocytes and macrophages, it has been demonstrated that 95% in the organism is PMN derived [18]. Hence MPO is considered as a valid marker of PMN activation. Therefore elevation of MPO plasma levels suggests that PMN activation is a particularly early event in the time course of AMI. Previous study [19] has shown that even when patients presented within the first 2 h after symptom onset, MPO levels were already elevated in comparison to patients with stable CAD. In contrast, however, routine markers of myocardial cell damage or coronary inflammation (i.e. hsCRP) were not increased. Funayama et al. had already proved that local MPO levels in the culprit artery may contribute to the NR phenomenon in patients with AMI [5] but did not provide evidence of mechanisms to explain their results. Furthermore, the NR was defined according to the final TIMI flow, which is far less accurate than CMR. In our study, CMR analysis provided additional information on the extent of MO, IS and ventricular remodeling, and allowed us to highlight for the first time the relationship between a high plasma level of MPO at the culprit lesion site and the enlargement of the IS after STEMI. Indeed, patients with high plasma MPO levels had a greater IS on the CMR done in the first 3 days after STEMI, and IS was still greater in these patients at 6 months after AMI. This association is strengthened by the strong correlation that we found between the troponin level, peak creatinine kinase and the plasma MPO level in the culprit artery. This was confirmed by our CMR data. In the present study, we provide evidence of 6 months persistence of microvascular obstruction in patients after reperfused AMI. Reffelmann et al. previously reported in rats that NR persists for 1 month after reperfusion and predicts worse scar thinning and infarct expansion [20]. Though we were not able to provide reliable scar thicknesses, the infarct size, expressed as a percentage of total LV mass, correlated inversely with systolic LV volumes (r-0.51, p = 0.04) [21]. Indeed, it has already been shown that plasma MPO levels were an independent predictor of myocardial infarction at two years in patients with AMI [22].

Considering the strong relation between MPO and systemic endothelial function, as a readout of vascular NO bioavailability [23,24] on the one hand and the robust data showing a markedly diminished vascular NO availability in AMI on the other hand [25,26], it might also be speculated that systemic activation of leukocytes, particularly via secretion of MPO, is one mechanism by which AMI leads to systemic endothelial dysfunction. To the best of our knowledge, our study provided new insights regarding the interaction between serum myeloperoxidase levels, endothelial dysfunction parameters (L-arginine, ADMA, SDMA) and ROS, and the impact of this interaction on clinical features including the microvascular obstruction infarct size and ventricular remodeling. First, we found that L-arginine plasma levels were significantly lower in the high-MPO group. That was unexpected, and we can hypotheses that MPO and its reactive products, including hypochlorous acid, may react with L-arginine [27], thus reducing the availability of substrate for nitric oxide synthase and forming chlorinated arginine species that inhibit the enzyme [28]. However, this is not a major reaction of HOCl as demonstrated by Pattison [29]. Myeloperoxidase and hypochlorous acid also reduce the availability of NADPH, an essential cofactor for nitric oxide synthase. Whereas we found no difference between our groups for the FORT test results, Brennan et al. provided strong data that myeloperoxidase is able to nitrate proteins via the production of reactive oxygen and nitrogen species such as peroxynitrite and nitrogen dioxide. Second, we found a trend towards higher SDMA levels in the high-MPO group: ADMA is an endogenous competitive inhibitor of all isoforms of NO synthases [30], and is now considered the most important regulator of the L-arginine/NO pathway in vivo. Our group previously reported the negative clinical impact of high admission ADMA in patients with MI [31]. Though SDMA, the stereoisomer of ADMA, is not a competitive inhibitor of NO synthases, high plasma concentrations of both ADMA and SDMA are associated with decreased brachial artery flow-mediated response. Myeloperoxidase-mediated endothelial dysfunction may be an important mechanistic link in the microvascular obstruction, since previous experimental data indicated that ADMA profoundly impairs nitric oxide synthesis in neutrophils, resulting in increased neutrophil adhesion to endothelial cells, superoxide generation, and the release of MPO. MPO appears to be a regulatory switch for ADMA bioavailability in inflammatory conditions such as MI [32]. We can hypothesize that MPO released at the culprit lesion site could destabilize culprit and non-culprit lesions and thus aggravate the microvascular obstruction.

## Study limitation

This study is certainly limited by its small sample size and further studies with larger numbers of patients are required to prove our hypothesis

## Conclusion

Our study provided data suggesting an interaction between MPO and the mechanisms of endothelial dysfunction. Microvascular obstruction after AMI was more severe, IS was greater and systolic LV remodeling more pronounced in the high-MPO group. Our present study may provide a new insights pathophysiology explanation for the adverse prognostic impact of NR over time.
